# Investigating PhD students’ wellbeing and mental health: towards evidence-led and innovative solutions

**DOI:** 10.3389/fpsyg.2026.1900506

**Published:** 2026-08-19

**Authors:** Riccardo Vecchio, Giansanto Mosconi, Martina Gandola, Andrea Amerio, Anna Odone, Health Mode On Investigators, Giovanni Albini, Chiara Barbati, Lavinia Barone, Mariagrazie Baroni, Paola Bertuccio, Laura Brunelli, Silvio Brusaferro, Anna Carli, Virginia Casigliani, Cristiano Crescentini, Elisa Diadema, Michele Emdin, Fabrizio Faggiano, Daniele Fedeli, Andrea Fossati, Serena Lecce, Emina Mehanović, Giansanto Mosconi, Anna Ogliari, Anna Odone, Francesca Pennucci, Filippo Quattrone, Alfredo Raglio, Caterina Rizzo, Emanuele Rossi, Valentina Tobia, Riccardo Vecchio, Giacomo Pietro Vigezzi, Federica Vigna-Taglianti, Elena Vivaldi, Luca Viviani, Patrizia Zeppegno, Marzia Zingarelli, Cesare Zizza

**Affiliations:** 1 Department of Public Health, Experimental and Forensic Medicine, University of Pavia, Pavia, Italy; 2 Medical Direction, Istituti Clinici Scientifici Maugeri IRCCS, Pavia, Italy; 3 IRCCS Mondino Foundation, Pavia, Italy; 4 Department of Brain and Behavioral Sciences, University of Pavia, Pavia, Italy; 5 Cognitive Neuropsychology Centre, ASST Grande Ospedale Metropolitano Niguarda, Milan, Italy; 6 NeuroMi, Milan Center for Neuroscience, Milan, Italy; 7 Department of Neuroscience, Rehabilitation, Ophthalmology, Genetics, Maternal and Child Health, Section of Psychiatry, University of Genoa, Genoa, Italy; 8 IRCCS Ospedale Policlinico San Martino, Genoa, Italy; 9 Medical Direction, Fondazione IRCCS Policlinico San Matteo, Pavia, Italy

**Keywords:** academic environment, graduate students, mental health, PhD students, wellbeing

## Abstract

PhD candidates face elevated risks of anxiety, depression, and burnout. Yet, mental health in doctoral education remains under-addressed and insufficiently supported by systematic data and interventions. In response, the Italian Ministry of University and Research launched PRO-BEN, a nationwide initiative aimed at investigating and promoting undergraduate and graduate student wellbeing. Within this framework, the “*Health Mode On*” project engages 10 universities and conservatories across Italy, targeting nearly 12,000 students, with a specific focus on doctoral candidates. As part of the assessment component, a survey adapted from the World Mental Health International College Student (WMH-ICS) initiative was expanded through a Delphi-informed process to include lifestyle-related domains. A pilot study conducted in July 2025 at the University of Pavia involved 352 PhD students (36.3% response rate). Preliminary findings showed a 12-month prevalence of 18.5% for mood disorders and 25.0% for anxiety disorders, highlighting a substantial burden of mental health conditions. By integrating epidemiological assessment with evidence-led interventions, training, and institutional engagement, the project aims to provide a scalable framework to support doctoral wellbeing.

## 1 Introduction

New evidence on the mental health crisis among early-career researchers draw attention to one of academia’s most pressing issues: the hidden burden of psychological distress affecting the next generation of researchers (Cilli et al., 2023).

Building on this perspective, recent reviews have further underscored the multifaceted nature of PhD students’ wellbeing and the urgent need for coordinated, evidence-informed efforts across individual, institutional, community, and policy levels (Mahsood et al., 2025).

Despite growing recognition of the importance of mental health in academic settings (Morris et al., 2021), doctoral researchers’ wellbeing remains a largely under-addressed issue. The transition from student to independent academic, intrinsic to the PhD journey, is characterized by uncertainty and stress, which may have lasting effects on aspiring researchers’ mental health, undermining higher education systems’ outputs and sustainability (Jovanović et al., 2019).

Over the past few years, several studies have documented the burden and determinants of psychological distress among PhD candidates (Martínez-García et al., 2024). Doctoral training, considered the highest educational attainment, is often accompanied by elevated levels of stress, pressure, isolation, and loneliness (Gonzalez-Aumatell et al., 2022).

The evidence is striking: PhD candidates are up to six times more likely to experience anxiety and depression compared to the general population (Evans et al., 2018), with higher risks persisting even when compared to age-comparable groups (Barry et al., 2018). In Belgium, 32% of doctoral candidates were reported at risk of developing mental health issues related to stress (Levecque et al., 2017). Similar findings emerged from surveys conducted in other countries. In Australia, a national questionnaire-based study found that 45% of PhD students reported moderate-to-severe depressive symptoms, and 36% were considered at risk of suicide (Mills et al., 2024). Moreover, a survey carried out in the Netherlands indicated that 47% of doctoral candidates were at risk of developing psychiatric disorders, while 39% exhibited severe symptoms of burnout (Promovendi Netwerk Nederland, 2020). Longitudinal data from Sweden further reported that PhD candidates’ use of psychiatric medications increased by 40% by their fifth year of study, compared to pre-enrolment levels (Schwaller, 2024).

Commonly cited barriers to wellbeing and mental health in this population include isolation, workload, peer pressure, lack of permanent employment, financial insecurity, and competitive academic environments (Huisman et al., 2002). Mental health stigma, normalization of distress, and fear of academic consequences discourage help-seeking (Ishikawa et al., 2023). The so-called impostor syndrome, which affects students who struggle to accept their own academic achievements, has been shown to correlate with reduced self-efficacy and lack of motivation (Forrester, 2021; Den Bakker et al., 2024). Social isolation, often linked to the siloed nature of doctoral research and limited peer interaction, has been widely reported by doctoral students (Jackman et al., 2023) with loneliness emerging as a strong predictor of key psychological outcomes, including depression, anxiety, and suicidality (Mills et al., 2024). Setting-specific variability in mental health outcomes and environmental factors is observed across academic departments and disciplines (Barreira and Bolotnyy, 2024). Moreover, intersectional vulnerabilities such as gender, including identifying as LGBTQ+ or non-binary and financial instability appear to further exacerbate mental health risks (Else, 2021; Woolston, 2019). The COVID-19 pandemic highlighted the mental health challenges faced by doctoral students, with one-third of respondents reporting post-traumatic stress symptoms across 12 U.S. public research universities during June–July 2020 (NSF RAPID, 2020; Paucsik et al., 2022).

Notably, candidate-supervisor relationships are consistently identified as both risk and protective factors (Petruzziello et al., 2026). Lack of support, unconstructive criticism, and imbalanced supervisory dynamics can intensify symptoms, while empathy and mentorship correlate with improved outcomes (Nature, 2019; Acker and Haque, 2015).

Despite this alarming evidence, many PhD students do not receive adequate support. University counselling services often lack resources, flexibility, or specificity to meet the unique needs of graduated communities (Jackman et al., 2023). Structural limitations include limited hours, long waiting times, financial barriers, and concerns over confidentiality (Woolston, 2019). Only a fraction of PhD students with clinical symptoms actively seek help (Byrom, 2018).

There is increasing consensus around the need for systemic interventions, including peer support networks, supervisor training, redesign of common spaces, structured mentorship, and proactive mental health screening using validated tools (Al Makhamreh and Stockley, 2019; Hall, 2024). A comprehensive review identified nine key factors affecting PhD wellbeing, ranging from supervision and department structures, to internal dimensions like self-worth, motivation, and academic identity (Sverdlik et al., 2018). Emerging evidence also supports the potential role of low-cost environmental and nature-based interventions, with a recent pilot study among graduate students finding that short-term exposure to forest environments significantly reduced negative emotional states and suggested that even unguided nature-based activities may represent scalable and accessible strategies for stress management in academic populations (Zhang et al., 2026).

A recent research paper has highlighted that digital support programmes have been shown to improve access to care by overcoming barriers such as fixed working hours and travel requirements. Their anonymous nature also may reduce the impact of stigma compared to in-person interventions, helping to mitigate structural and attitudinal barriers and bridge gaps in care provision. Using a mixed-methods pre–post design, the authors found that an asynchronous webinar significantly improved attitudes towards help-seeking among PhD students (Hardy et al., 2026). They also reported that 21.8% of participants met the clinical threshold for moderate to severe levels of depression and anxiety. Moreover, as part of the so-called “Dragonfly Mental Health Initiative,” the screening of a short film featuring interviews with academic professionals discussing their own mental health challenges was found to reduce stigma around the topic for 92% of viewers, including faculty, postdoctoral researchers, graduate students, and university staff members (Schwaller, 2025; Devendorf et al., 2025).

Yet, not only scant evidence is available from large-scale, setting-specific representative data on PhD students mental health (Evans et al., 2018), but also limited intervention studies evaluated the impact of prevention or care programmes. In addition, most studies do not differentiate between different levels of graduate education, rely on limited samples and cross-sectional designs, and rarely address mental health disorders (Hazell et al., 2020).

In Italy, the lack of standardized epidemiological data and coordinated surveillance initiatives may limit the ability to comprehensively assess the mental health needs of doctoral students and to develop evidence-informed institutional strategies.

Generating standardized cross-sectional data is therefore an essential first step to quantify the burden of mental health disorders, identify population subgroups at greater risk, and provide the evidence base needed to design preventive and supportive interventions. Building on these data, longitudinal surveillance and repeated assessments can subsequently monitor trends over time, evaluate the effectiveness of institutional actions, and support the continuous improvement of mental health promotion strategies within doctoral education. This evidence is essential not only to quantify the burden of mental health disorders but also to identify modifiable determinants, prioritize institutional actions, and establish the foundations for evaluating future mental health promotion strategies within doctoral education.

## 2 Method

To address this gap, the Italian Ministry of University and Research launched the nation-wide initiative PRO-BEN (Italian Ministry of University and Research, 2024) funded between 2023 and 2025 with over €60 million aimed at supporting Italian universities and conservatories in conducting comprehensive analysis of mental health burden and associated issues, identifying both latent and expressed mental health needs within the academic community, planning, implementing and monitoring interventions, and ultimately creating an all-encompassing “Healthy Campus” ecosystem to enhance overall wellbeing among students, including doctoral students. Within the PRO-BEN initiative, the “*Health Mode On*” project brings together 10 universities across Italy (the University of Pavia as project leader, the “Antonio Vivaldi” Conservatory of Music in Alessandria, the “Giuseppe Verdi” Conservatory of Music in Milan, the “Jacopo Tomadini” Conservatory of Music in Udine, the “Pietro Mascagni” Conservatory of Music in Livorno, the Sant’Anna School of Advanced Studies in Pisa, the “Amedeo Avogadro” University, the University of Udine; the University of Pisa, and the Vita-Salute San Raffaele University). The consortium includes experts in epidemiology, psychiatry, psychology, education sciences, and public health, targeting a total of nearly 12,000 undergraduate and graduate students. Within this broader framework, a specific focus is placed on PhD candidates, who represent a high-risk and under-investigated population. The project is articulated in four key components: (i) the assessment, that implies mapping the prevalence and severity of psychological and emotional distress among university students while also exploring lifestyle factors and access to health services; (ii) evidence-led interventions focusing on identifying and implementing integrated models to promote student wellbeing and connecting existing university counselling services with public mental health services; (iii) training for academic staff; (iv) communication and dissemination of insights, experiences and lessons learned.

Within the assessment component, a comprehensive survey was designed and adapted from the model of the World Mental Health International College Student (WMH-ICS) initiative, coordinated by the Harvard Medical School in collaboration with World Health Organization (WHO) (Barreira and Bolotnyy, 2024). The WMH-ICS survey instrument includes validated Composite International Diagnostic Interview Screening Scales (CIDI-SC) designed to estimate the lifetime and 12-month prevalence of common DSM-5 mental disorders while ensuring international comparability across higher education settings. Specifically, it assesses major depressive disorder (MDD), broad bipolar spectrum disorder (BPD), generalized anxiety disorder (GAD), panic disorder (PD), attention-deficit/hyperactivity disorder (ADHD), and drug use disorder (DUD) (Vigezzi et al., 2026; Mak et al., 2022).

To complement the WMH-ICS core assessment, we developed an additional questionnaire through a structured e-Delphi process involving experts from epidemiology, psychiatry, psychology, public health, and health promotion within the *Health Mode On* consortium (Mosconi and Health Mode On Investigators, 2026). The additional modules were specifically designed to preserve the international comparability of the WMH-ICS framework while expanding the assessment to capture lifestyle, psychosocial, and contextual determinants of mental wellbeing relevant to the Italian university setting. The additional research domains included lifestyle, psychological, and contextual domains relevant to student wellbeing, including sleep, physical activity, dietary habits, loneliness, academic stress, gambling, gaming, social media use, housing conditions, financial strain, discrimination, and access to support services (Mosconi et al., 2025).

A pilot study was launched in July 2025 at the University of Pavia as part of the broader *Health Mode On* initiative, specifically targeting the doctoral students. The survey, available in Italian and English, was distributed to all PhD candidates enrolled at the University of Pavia (*n* = 970). PhD students were invited to complete the online survey on a voluntary basis between July and September 2025.

## 3 Preliminary results

Overall, 352 PhD students completed the survey, corresponding to a response rate of 36.3%.

The study population included a balanced representation of male and female doctoral students. Participants had a median age of approximately 29 years. All the three main academic areas of the University of Pavia were represented: Science and Technology, Life Sciences, and Humanities and Social Sciences.

Preliminary findings revealed that three out of five met the CIDI-SC criteria for a mental disorder during the previous year. In particular, 12-month prevalence was 18.5% for mood disorders and 25.0% for anxiety disorders, highlighting a substantial burden of mental health conditions among doctoral candidates.

The prevalence of mental disorders was higher among female than male doctoral students, whereas no significant differences emerged across age groups or nationality.

These estimates are broadly consistent with previous international evidence documenting elevated rates of depression, anxiety, and psychological distress among PhD students and early-career researchers.

In addition to the preliminary prevalence estimates, the integrated assessment framework captured behavioural, lifestyle, psychosocial, socioeconomic, and academic determinants of mental health, providing the basis for future analyses aimed at identifying determinants and modifiable risk factors while informing targeted interventions.

## 4 Discussion

To the best of our knowledge, the pilot represented the first application of the WMH-ICS assessment to an Italian doctoral student population. The preliminary findings confirm the relevant mental health distress among doctoral researchers, consistently with previous international evidence (Hardy et al., 2026). Although derived from a single institution, they provide an initial epidemiological baseline and reinforce the need for systematic, standardized surveillance of doctoral students’ mental health across Italian higher education institutions.

Indeed, beyond the prevalence estimates themselves, the pilot phase provided important insights regarding the implementation of a large-scale mental health surveillance system within academic environments. The high response rate supports the feasibility of administering a comprehensive mental health assessment framework within a doctoral education setting.

The *Health Mode On* project was conceived not only as an assessment initiative but also as a platform for intervention, training, and institutional change. The adoption of a standardized and internationally comparable framework, integrated with lifestyle, behavioural, and contextual dimensions identified through a structured e-Delphi process, will offer the opportunity to generate actionable evidence on both mental health outcomes and their determinants. By enabling the identification of modifiable determinants of mental health and academic functioning, the initiative aims to support universities in prioritizing preventive actions, tailoring support services, and evaluating the impact of institutional interventions over time.

Moreover, the multicentre consortium enables this surveillance framework to be extended across diverse academic settings while generating more robust national estimates and fostering collaboration among public health professionals, psychologists, psychiatrists, educators, and other stakeholders involved in student wellbeing.

While the project progresses, the integration of standardized epidemiological surveillance with multidisciplinary expertise will provide an unique opportunity to better understand doctoral students’ mental health needs, identify priorities for intervention, and support the development and evaluation of evidence-informed policies and health promotion initiatives within Italian higher education.

As the “practice of wellbeing” has been proposed as a foundational skill for the next generation of future researchers (Valks et al., 2023), we do believe that investing in the mental health of PhD candidates is not only an individual matter, but a cornerstone for the future of academia and innovation.

## Data Availability

The data that support the findings of this study are available from the corresponding author upon reasonable request. Requests to access the datasets should be directed to anna.odone@unipv.it.

## Group members of Health Mode On Investigators

Giovanni Albini (Conservatory of Music Antonio Vivaldi, Alessandria), Chiara Barbati (Department of Public Health, Experimental and Forensic Medicine, University of Pavia, Pavia), Lavinia Barone (Department of Brain and Behavioral Sciences, University of Pavia, Pavia), Mariagrazie Baroni (Conservatory of Music Antonio Vivaldi, Alessandria), Paola Bertuccio (Department of Public Health, Experimental and Forensic Medicine, University of Pavia, Pavia), Laura Brunelli (Department of Medicine, University of Udine, Udine; Accreditation, Quality, and Clinical Risk Unit, Friuli Centrale Healthcare University Trust, Udine), Silvio Brusaferro (Department of Medicine, University of Udine, Udine, Italy; Accreditation, Quality, and Clinical Risk Unit, Friuli Centrale Healthcare University Trust, Udine), Anna Carli (Conservatory of Music Rinaldo Franci, Siena), Virginia Casigliani (Department of Translational Research and of New Surgical and Medical Technologies, University of Pisa, Pisa), Cristiano Crescentini (Department of Languages and Literatures, Communication, Education and Society, University of Udine, Udine), Elisa Diadema (Department of Translational Research and of New Surgical and Medical Technologies, University of Pisa, Pisa), Michele Emdin (Interdisciplinary Research Center for Health Science, Sant’Anna School of Advanced Studies, Pisa), Fabrizio Faggiano (Department of Sustainable Development and Ecological Transition, University of Eastern Piedmont, Vercelli, Italy; Osservatorio Epidemiologico, ASL Vercelli, Vercelli), Daniele Fedeli (Department of Languages and Literatures, Communication, Education and Society, University of Udine, Udine), Andrea Fossati (School of Psychology, Vita-Salute San Raffaele University, Milan; Clinical Psychology and Psychotherapy Unit, San Raffaele Turro Hospital, Milan), Serena Lecce (Department of Brain and Behavioural Sciences, University of Pavia, Pavia), Emina Mehanović (Department of Sustainable Development and Ecological Transition, University of Eastern Piedmont, Vercelli), Giansanto Mosconi (Department of Public Health, Experimental and Forensic Medicine, University of Pavia, Pavia), Anna Ogliari (School of Psychology, Vita-Salute San Raffaele University, Milan), Anna Odone (Department of Public Health, Experimental and Forensic Medicine, University of Pavia, Pavia; Medical Direction, Fondazione IRCCS Policlinico San Matteo, Pavia), Francesca Pennucci (Interdisciplinary Research Center for Health Science, Sant’Anna School of Advanced Studies, Pisa), Filippo Quattrone (Interdisciplinary Research Center for Health Science, Sant’Anna School of Advanced Studies, Pisa), Alfredo Raglio (Conservatory of Music Giuseppe Verdi, Milan; Istituti Clinici Scientifici Maugeri IRCCS, Pavia), Caterina Rizzo (Department of Translational Research and of New Surgical and Medical Technologies, University of Pisa, Pisa), Emanuele Rossi (Conservatory of Music Pietro Mascagni, Livorno; Sant’Anna School of Advanced Studies, Pisa), Valentina Tobia (School of Psychology, Vita-Salute San Raffaele University, Milan), Riccardo Vecchio (Department of Public Health, Experimental and Forensic Medicine, University of Pavia, Pavia), Giacomo Pietro Vigezzi (Department of Public Health, Experimental and Forensic Medicine, University of Pavia, Pavia), Federica Vigna-Taglianti (Department of Translational Medicine, University of Eastern Piedmont, Novara, Italy), Elena Vivaldi (Interdisciplinary Research Center for Health Science and Dirpolis Institute, Sant’Anna School of Advanced Studies, Pisa), Luca Viviani (Department of Public Health, Experimental and Forensic Medicine, University of Pavia, Pavia), Patrizia Zeppegno (Department of Translational Medicine, University of Eastern Piedmont, Novara, Italy, SC Psichiatria AOU Maggiore della Carità, Novara), Marzia Zingarelli (Conservatory of Music Antonio Vivaldi, Alessandria), Cesare Zizza (Department of Humanities, University of Pavia).
